# Diagnostic and prognostic value of miR-486-5p, miR-451a, miR-21-5p and monocyte to high-density lipoprotein cholesterol ratio in patients with acute myocardial infarction

**DOI:** 10.1007/s00380-022-02172-2

**Published:** 2022-10-10

**Authors:** Liwenjing Xu, Lu Tian, Zhenrong Yan, Jia Wang, Tingyun Xue, Qiyu Sun

**Affiliations:** Clinical Laboratory, The Affiliated Hospital of Chengde Medical University, Chengde, Hebei China

**Keywords:** Circulating miRNAs, MHR, Acute myocardial infarction, PCI, Biomarkers

## Abstract

Acute myocardial infarction (AMI) is one of the most serious complications of coronary heart disease. Although morbidity and mortality have been decreasing year by year, acute coronary syndrome still has a high mortality rate and disability rate. To search for accurate and effective biomarkers, we explore the diagnostic and prognostic value of microRNAs (miRNAs) and the monocyte to high-density lipoprotein cholesterol ratio (MHR) in patients with AMI. By referring to the relevant literature, miR-486-5p, miR-451a and miR-21-5p were reportedly altered in the blood of patients with ischemic heart disease. These miRNAs were selected and validated in 40 AMI patients, 22 unstable angina pectoris (UAP) and 22 healthy groups (HC) by real-time fluorescent quantitative polymerase chain reaction (RT-qPCR). All patients with AMI underwent primary percutaneous coronary intervention (PCI) and were followed up 3 months after the operation. MHR and miR-451a expression were markedly elevated in plasma samples of AMI patients compared with the UAP and HC groups, but the expressions of miR-486-5p and miR-21-5p were significantly decreased. The expression level of miRNA-451a increased gradually among the three groups (*p* < 0.05). However, the expression of miRNA-21-5p showed a downward trend (*p* < 0.05). More importantly, MHR was significantly different before and after PCI in AMI patients (*p* < 0.05). Receiver operating characteristic (ROC) analysis indicated that MHR, miR-486-5p, miR-451a and miR-21-5p could diagnose and predict AMI. MiR-451a was a more reliable biomarker for AMI diagnosis among these miRNAs. Moreover, the combination of MHR and miRNAs had higher diagnostic value for AMI. We further demonstrated that miR-21-5p had a strong predictive ability for the occurrence of major adverse cardiovascular events (MACE) after 3 months. The results showed that circulating miR-486-5p, miR-451a, miR-21-5p and MHR may play critical roles in the early phase of AMI, and may be used as potential predictors for AMI diagnosis. Importantly, miR-451a was a more reliable biomarker in diagnosing AMI patients. Circulating miR-21-5p may be used as a predictor of MACE occurrence.

## Introduction

At present, cardiovascular disease (CVD) is the leading cause of death worldwide, causing immense health and economic burdens, of which acute myocardial infarction (AMI) has the greatest impact [1]. More than one-half of the deaths in AMI patients before they reach the hospital for treatment [2]. Therefore, early diagnosis and effective treatment are of great significance for reducing myocardial cell damage, reducing mortality and improving patient prognosis. As one of myocardial injury biomarkers, cardiac troponin (cTn) is widely used in clinical practice. However, it takes 3–4 h for the troponin level to increase after the occurrence of AMI, and can neither indicate the potential damage mechanism nor distinguish between ischemic and non-ischemic. In addition, false positive results of elevated cTn could associate with disease such as acute pulmonary embolism, heart failure (HF), chronic kidney diseases, myocarditis, and arrhythmia [3]. Therefore, accurate molecular biomarkers for the diagnosis and treatment of AMI in the non-symptom stage are urgently need to improve the prognosis.

MicroRNAs (miRNAs) are a kind of small noncoding single-stranded RNA that are approximately 21–23 nucleotides in length. The main function of miRNAs is to inhibit the translation, degradation and deadenylation of target genes by binding to the 3′ untranslated region (UTR) of its downstream target gene mRNA [4]. Furthermore, endogenous plasma miRNAs are strongly conserved during evolution and can resist plasma RNase activity. Prior reports have suggested that miRNAs are present in clinical samples of plasma and serum in a remarkably stable form and may serve as reliable blood-based biomarkers [5, 6]. The presence of miRNAs is also associated with specific cardiovascular diseases, which are important for the identification of different cardiovascular diseases [7].

MiR-486 is a muscle-enriched miRNA encoded by the intron 40 of the *Ankyrin-1* (ANK-1) gene [8]. Studies showed that over-expression of miR-486-5p reduced cardiomyocyte apoptosis and improved cardiac function through inhibition of PTEN and activation of the PI3K/AKT signaling pathway [9]. MiR-451 is located on chromosome 17q11.2 [10]. Former studies have shown that miR-451 may induce cardioprotection in anoxia/reoxygenation (A/R) injury by attenuating high mobility group box 1(HMGB1) expression [11]. MiR-486-5p and miR-451a were predicted to regulate genes within the atherosclerosis pathway during the previous gene miRNA interaction network analysis, and miR-486-5p and miR-451a whose role in atherosclerosis had been validated [12]. Moreover, miR-486-5p and miR-451a were reportedly altered in the blood of patients with ischemic heart disease (IHD) [13]. Due to atherosclerosis is the main cause of AMI, stroke and UAP, miR-486-5p and miR-451a are selected for validation by qPCR with patients in AMI. MiR-21 was later shown to be a cardiac fibroblast-derived miRNA. The diagnostic effect of serum miR-21 of AMI is still controversial. Someone measured levels of miR-21 in AMI. They observed a higher level of serum miR-21in the AMI group [14]. Nevertheless, some studies also showed that the expression of miR-21 was decreased, and it was a cardio-protective miRNA in AMI [15]. Based on the above analysis, the role of these miRNAs in AMI s requires a further investigation.

In recent years, many studies have suggested that MHR is an independent predictor of coronary artery stenosis. Moreover, it was reported that the rates of in hospital mortality and major adverse cardiac events (MACE) were higher in patients with STEMI after PCI who had higher MHR values compared with those with lower MHR values [16]. We further validate the value of MHR in combination with miRNAs with patients in AMI.

Based on the above, we aim to analyze the expression of miR-486-5p, miR-451a, miR-21-5p and MHR in AMI patients before and after PCI by comparing with healthy controls, so as to provide strategies for AMI diagnosis and survival prediction.

## Materials and methods

### Clinical samples

#### Clinical data of patients

The study included 40 AMI patients and 44 non-MI patients (22UAP and 22 HC) from October 2019 to August 2020 at Affiliated Hospital of Chengde Medical College. Performance of the study was according to the principles of the Declaration of Helsinki and approved by the Ethics Committee of the Affiliated Hospital of Chengde Medical College. All patients signed an informed consent form.

#### Inclusion of exclusion criteria

The inclusion criteria were as follows: patients were admitted to the hospital within 6 h. All AMI patients were diagnosed for the first time, and they underwent coronary angiography and PCI. The diagnostic criteria for AMI were based on the 2018 ESC/ACCF/AHA/WHF Fourth universal definition of myocardial infarction [17]. UAP was diagnosed with reference to the criteria recommended by Chacko KA [18]. The HC was healthy subjects who had not been diagnosed with cardiovascular disease.

The exclusion criteria were as follows: including previous history of cardiac diseases (AMI, heart failure, arrhythmia or cardiomyopathy), malignant tumors, acute infectious diseases, liver and kidney dysfunction, autoimmune diseases, surgery in the previous months, severe infection.

#### Sample collection and storage

The venous blood samples from subjects were collected. In AMI group, the blood was collected immediately after the admission of the patient to the hospital and 1 h after PCI. The control group was collected from patients who received physical examination and diagnosed with UAP. The collected blood samples were placed in EDTA anticoagulant tubes (5 mL). All blood samples were centrifuged at 3000 rpm for 10 min to separate plasma from red blood cells. Next, the plasma samples were transferred into DNase/RNase free tubes and centrifuged at 12,000 rpm for 15 min to remove platelets from the samples. Finally, the plasma was removed to an RNase/DNase-free tube and stored at − 80 °C until use.

#### Follow-up and study

Participants with AMI were followed for 3 months by telephone and inpatient records. The primary endpoint was the occurrence of MACE, consisting of composite cardiac death, recurrent AMI, repeat revascularization, unstable angina, stroke or hospitalization due to acute heart failure.

### Quantitative real-time PCR (RT-qPCR)

#### Total RNA extraction

An miRcute serum/plasma miRNA isolation kit (TIANGEN, China) was used to extract total RNA according to the manufacturer's instructions. The synthesized miRNA cel-miR-39 (RiboBio Co Guangdong, China) was added as an internal control to each clinical sample and made into the final concentration of 10^−4^ pmol/L. The Biospec-mini UV spectrophotometer was applied to verify the quality of isolated total RNAs from 5 mL plasma. U6 was used as the endogenous controls of miR-486 expression when it concerns the expression of miRNA in rats and H9c2 cells.

#### miRNA polyadenylation and reverse transcription

The cDNA was generated by using BulgeLoopTM miRNA qRT-PCR starter kit (RiboBio Co Guangdong, China) according to the manufacture’s protocol. The reaction was performed in a thermocycler. The reverse transcription process was carried out at 42 ℃ for 60 min and then at 70 ℃ for 10 min. At last, the cDNA was stored at − 20 ℃ until use.

#### miRNA validation

The PCR was carried out according to SYBR qPCR Kit (RiboBio Co, Guangdong, China). The final reaction volume was 20 μL of reaction and was performed on a Roche Cobas z480 detection system (Roche Molecular Diagnostics). The PCR conditions were as follows: pre-denaturation at 95 ℃ for 10 min, denaturation at 95 ℃ for 2 s, annealing at 60 ℃ for 30 s and extending 70 ℃ for 10 s, with a total of 40 cycles. Finally, the dissociation and melting curves were produced to verify amplification specificity. *Caenorhabditis elegans* miRNA (cel-miR-39) was used as the normalization control. The relative expression scores of miRNAs were obtained directly from a real-time fluorescent quantitative PCR instrument, and the $2^{{ - \Delta \Delta C_{{\text{t}}} }}$ method was used to analyze the relative expression level of miRNAs. The sequence data of the miRNAs can be obtained from the website (http://www.mirbase.org/).

### Animals and cardiac I/R injury model

Male Sprague–Dawley (SD) rats were purchased from Beijing HFK bioscience Co., Ltd (Beijing, China) and maintained in the Medical Experimental Animal Center of Affiliated Hospital of Chengde Medical University (Hebei, China). All animal experimental procedures were approved by the Animal Care and Use Committee of Chengde Medical University and conformed to the Guide for the Care and Use of Laboratory Animals. The rats were anesthetized and connected to ventilators after endotracheal intubation. The AMI group were operated with ligating the left coronary artery while the sham-operated group only received thoracotomy without coronary artery ligation.

### Culture and treatment of H9c2 cell

The rat heart myoblast cells H9c2 were purchased from Procell Life Science&Technology Co., Ltd (Wuhan, China). All cells were cultured in Dulbecco’s modified Eagle’s medium (DMEM, Gibco, Carlsbad, USA) containing 4.5 g/L glucose supplemented with fetal bovine serum (FBS) and placed in a 37 °C incubator containing 5% CO_2_. To mimic cardiac hypoxia injury in vitro, H9c2 cells were cultured in hypoxic incubator with the atmosphere of 1% O_2_ and 99% CO_2_.

### Bioinformatic analysis of miRNAs

The target prediction of miRNA was performed using six online databases (TargetScan, starBase, miRDB, miRWalk, PITA and DIANA-TarBase). The candidate target genes were identified when the genes involved in at least three predictive programs. Further, GO and KEGG analyses were performed on the target genes.

### Statistical analysis

For descriptive purposes, the quantitative variables were presented as means ± standard deviations (SD). All continuous variables were checked using the Kolmogorov–Smirnov (K–S) test. The count data were assessed by Chi-square test (*χ*^2^). The normally distributed data were compared by independent sample *t* test. One-way ANOVA was used to test for differences more than three groups. The nonparametric test was used for the nonnormally distributed data. Spearman rank correlations were used to analyze the association of miRNAs expression levels among each other and with clinical variables. The combination among miRNAs were assessed using logistic regression. Receiver operating characteristic (ROC) curves and the area under the ROC curves (AUC) further analyzed the diagnostic values of the selected miRNAs. Statistical tests were performed using GraphPad Prism 8.0 (GraphPad Software, San Diego, CA, United States) and SPSS 28.0 software (IBM Corp., Armonk, NY). All statistical tests were two-tailed, and *p* < 0.05 indicated a statistically significant difference.

## Results

### Clinical data of the study population

The baseline characteristics of 40 AMI patients and 44 control subjects (22 UAP and 22 HC) were summarized in Table 1. The result showed that there were statistical differences in total cholesterol levels (TC), low-density lipoprotein (LDL-C), blood glucose (GLU), white blood cell count (WBC), creatine kinase-MB (CK-MB), cardiac troponin I (cTnI), monocytes (M), lymphocytes (L), neutrophils (N) in the AMI and non-MI groups (*p* < 0.05). Clinical variables such as TC, LDL-C, GLU, WBC, CK-MB, cTnI, M, L, N were increased (*p* < 0.05) in the AMI patients compared with non-MI group (Table 2).

**Table 1:** Baseline characteristics of all patients

Variables	AMI group (*n* = 40)	UAP group (*n* = 22)	HC group (*n* = 22)	*p*-value
Male/female (*n*/*n*)	30/10	14/8	13/9	0.253^a^
Age (years)	55.65 ± 11.36	61.86 ± 9.45	51.86 ± 12.50	0.015^b^
BMI (kg/m^2^)	26.19 ± 3.65	26.61 ± 4.21	25.45 ± 2.46	0.551^b^
Smoking history (%)	75.00%	59.09%	50.00%	0.122^a^
Hypertension (%)	75.00%	68.18%	40.90%	0.025^a^
SBP (mmHg)	158.18 ± 23.43	165.73 ± 41.05	140.68 ± 19.24	0.024^b^
DBP (mmHg)	87.83 ± 15.62	93.95 ± 20.58	79.09 ± 9.31	0.019^c^
Diabetes (%)	30.00%	22.73%	13.64%	0.347^a^
Killip class at admission ≥ II (%)	7.50%	0	0	0.181^a^
TC (mmol/L)	4.76 ± 0.93	4.29 ± 1.10	4.68 ± 1.38	0.036^c^
TG (mmol/L)	1.90 ± 1.48	1.92 ± 1.13	1.80 ± 1.18	0.606^c^
LDL (mmol/L)	2.84 ± 0.92	2.30 ± 0.97	2.58 ± 0.59	0.010^c^
HDL (mmol/L)	1.06 ± 0.28	1.02 ± 0.19	1.23 ± 0.35	0.037^b^
GLU (mmol/L)	9.25 ± 4.47	7.41 ± 2.21	5.56 ± 1.11	0.000^c^
WBC (× 10^9^/L)	11.68 ± 2.88	7.32 ± 2.62	6.27 ± 1.41	0.000^c^
PLT (10^9^/L)	235.35 ± 52.79	230.14 ± 68.91	246.27 ± 44.93	0.490^c^
Cr (µmol/L)	61.07 ± 16.13	64.27 ± 11.51	62.45 ± 11.26	0.685^b^
CK-MB (U/L)	150.42 ± 156.42	20.31 ± 29.43	9.40 ± 3.67	0.000^c^
cTnI (ng/mL)	12.28 ± 12.29	0.07 ± 0.07	0.05 ± 0.00	0.000^c^
M (× 10^9^/L)	0.55 ± 0.25	0.47 ± 0.24	0.36 ± 0.10	0.005^c^
L (× 10^9^/L)	1.83 ± 1.09	1.81 ± 0.47	2.29 ± 0.52	0.004^c^
N (× 10^9^/L)	8.88 ± 3.11	5.10 ± 3.56	3.23 ± 1.21	0.000^c^

Data are shown as the mean ± SD

*BMI* body mass index, *SBP* systolic blood pressure, *DBP* diastolic blood pressure, *TC* total cholesterol, *TG* triglyceride, *LDL* low-density lipoprotein, *HDL* high-density lipoprotein, *Glu* blood glucose, *WBC* white blood cell count, *PLT* platelet count, *Cr* creatinine, *CK-MB* creatine kinase-MB, *cTnI* cardiac troponin I, *N* neutrophile granulocyte, *L* lymphocyte, *M* monocyte

^a^Chi-squared test

^b^One-way ANOVA

^c^Kruskal–Wallis *H* test

**Table 2:** Analysis clinical characteristics of the AMI group and non-MI group (non-MI)

Variables	AMI group (*n* = 40)	Non-MI group (*n* = 44)	*p*-value
Male/female (*n*/*n*)	30/10	24/20	0.097^a^
Age (years)	55.65 ± 11.36	56.86 ± 12.15	0.638^b^
BMI (kg/m^2^)	26.19 ± 3.65	26.03 ± 3.52	0.579^c^
Smoking history (%)	75.00%	54.50%	0.051^a^
Hypertension (%)	75.00%	54.50%	0.051^a^
SBP (mmHg)	158.18 ± 23.43	153.20 ± 34.12	0.201^c^
DBP (mmHg)	87.83 ± 15.62	86.52 ± 17.49	0.615^c^
Diabetes (%)	30.00%	18.18%	0.204^a^
Killip class at admission ≥ II (%)	7.50%	0	0.064^a^
TC (mmol/L)	4.76 ± 0.93	4.48 ± 1.25	0.018^c^
TG (mmol/L)	1.90 ± 1.48	1.86 ± 1.14	0.622^c^
LDL (mmol/L)	2.84 ± 0.92	2.44 ± 0.81	0.038^b^
HDL (mmol/L)	1.06 ± 0.28	1.12 ± 0.30	0.361^c^
GLU (mmol/L)	9.25 ± 4.47	6.49 ± 1.97	0.00^c^
WBC (× 10^9^/L)	11.68 ± 2.88	6.80 ± 2.15	0.000^c^
PLT (10^9^/L)	235.35 ± 52.79	238.20 ± 58.06	0.670^c^
Cr (µmol/L)	61.07 ± 16.13	63.36 ± 11.29	0.457^b^
CK-MB (U/L)	150.42 ± 156.42	14.85 ± 21.45	0.000^c^
cTnI (ng/mL)	12.28 ± 12.29	0.06 ± 0.05	0.000^c^
M (× 10^9^/L)	0.55 ± 0.25	0.42 ± 0.19	0.003^c^
L (× 10^9^/L)	1.83 ± 1.09	2.05 ± 0.55	0.021^c^
N (× 10^9^/L)	8.88 ± 3.11	4.16 ± 2.79	0.000^c^

^a^Chi-squared test

^b^Student’s *t* test

^c^Mann–Whitney *U* test

### Expression of MHR, miR-486-5p, miR-451a, miR-21-5p in different groups

**Fig. 1:**
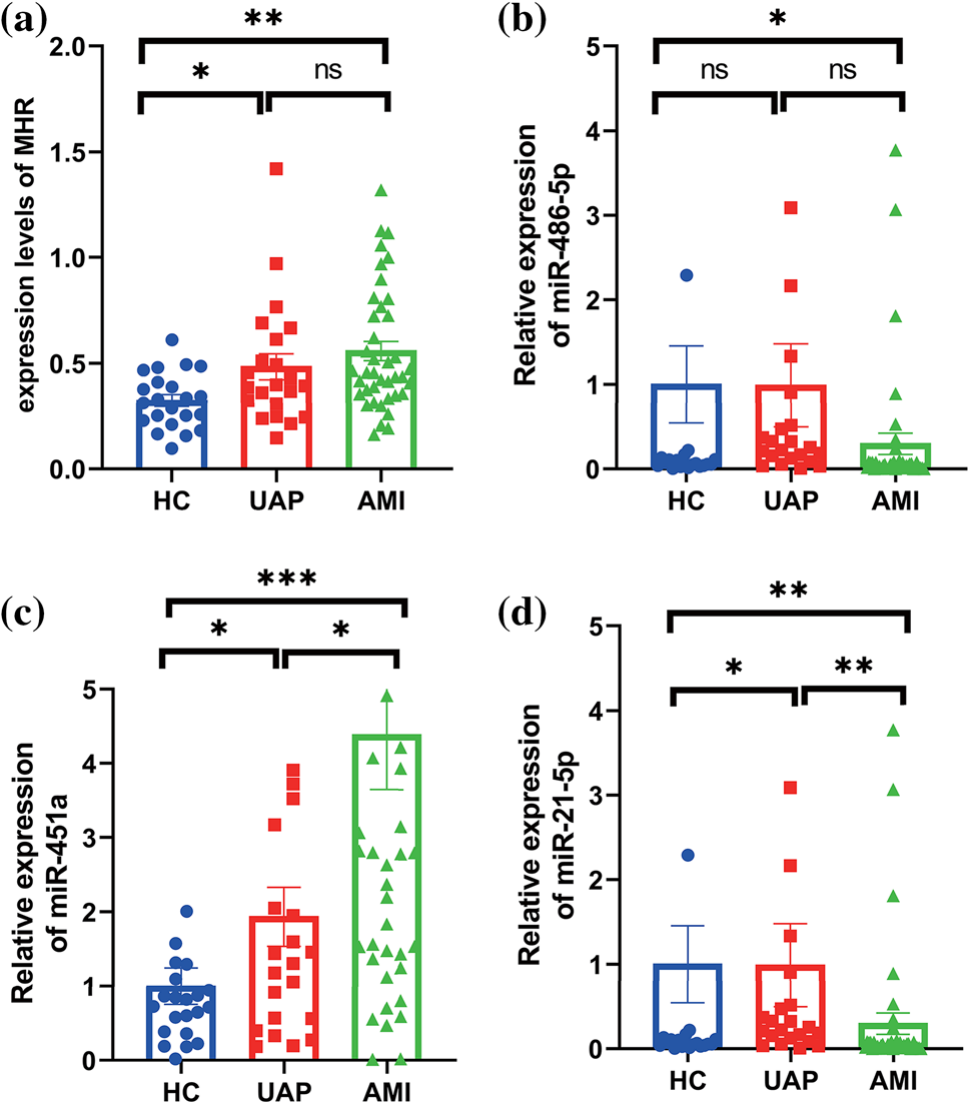
Expression levels of MHR (**a**), miR-486-5p (**b**), miR-451a (**c**) and miR-21-5p (**d**) in samples among three groups (*p < 0.05, **p < 0.01, ***p < 0.001)

**Fig. 2:**
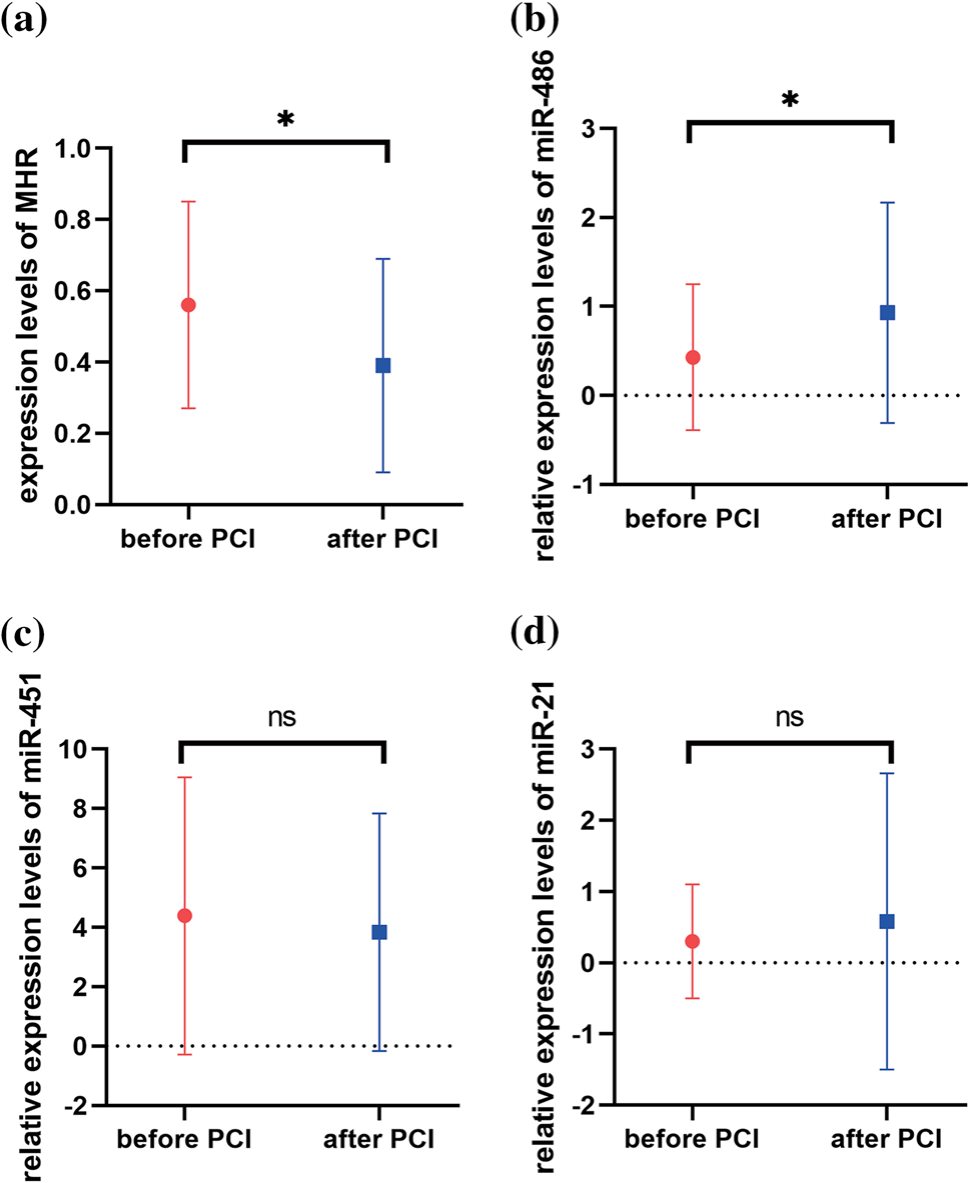
The expression levels of MHR (**a**), miR-486-5p (**b**), miR-451a (**c**) and miR-21-5p (**d**) in patients with AMI (including before and after PCI) and non-MI subjects (*p < 0.05, **p < 0.01, ***p < 0.001)

The results showed that the expressions of MHR, miR-451a and miR-21-5p were significantly different among the three groups (AMI, UAP and HC) (Table 3). The expression levels of MHR and miR-451a significantly increased in AMI compared with the UAP and HC groups. Contrary to the expression levels of the MHR and miR-451a, miR-486-5p and miR-21-5p were significantly decreased in AMI patients (Fig. 1). The expression of MHR was not statistically significant in AMI and UAP patients (Fig. 1a). However, miR-451a was significantly higher in UAP patients than the HC group (Fig. 1c). The level of miR-486-5p was significantly lower in AMI patients than HC group, but it was not significant difference in the AMI group compared with the UAP group. Moreover, miR-486-5p was also no significant difference between the UAP group and the HC group (Fig. 1b). We further validated the levels of MHR, miR-486-5p, miR-451a and miR-21-5p before PCI and after PCI in patients with AMI (Fig. 2). Compared with before PCI, the expression of MHR was significantly decreased and the expression of miR-486-5p was increased significantly after PCI. However, the expression miR-451a and miR-21-5p were no significant difference between before PCI and after PCI (*p* > 0.05).

**Table 3:** The expression levels of MHR and miRNAs in different groups

Variables	AMI group (*n* = 40)	UAP group (*n* = 22)	HC group (*n* = 22)	*p*-value
MHR				
Pre-PCI	0.56 ± 0.29	0.48 ± 0.29	0.32 ± 0.13	0.002^a^
Post-PCI	0.39 ± 0.30	–	–	< 0.05^b^
miR-486-5p				
Pre-PCI	0.43 ± 0.82	0.73 ± 1.08	1 ± 1.18	0.079^a^
Post-PCI	0.93 ± 1.24	–	–	0.034^b^
miR-451a				
Pre-PCI	4.39 ± 4.67	1.93 ± 1.86	1 ± 1.16	< 0.001^a^
Post-PCI	3.83 ± 4.00	–	–	0.823^b^
miR-21-5p				
Pre-PCI	0.30 ± 0.80	0.99 ± 2.31	1 ± 2.14	< 0.001^a^
Post-PCI	0.58 ± 2.08	–	–	1.000^b^

^a^Kruskal–Wallis test

^b^Wilcoxon test

### The diagnostic value of the candidate miRNAs in AMI

**Fig. 3:**
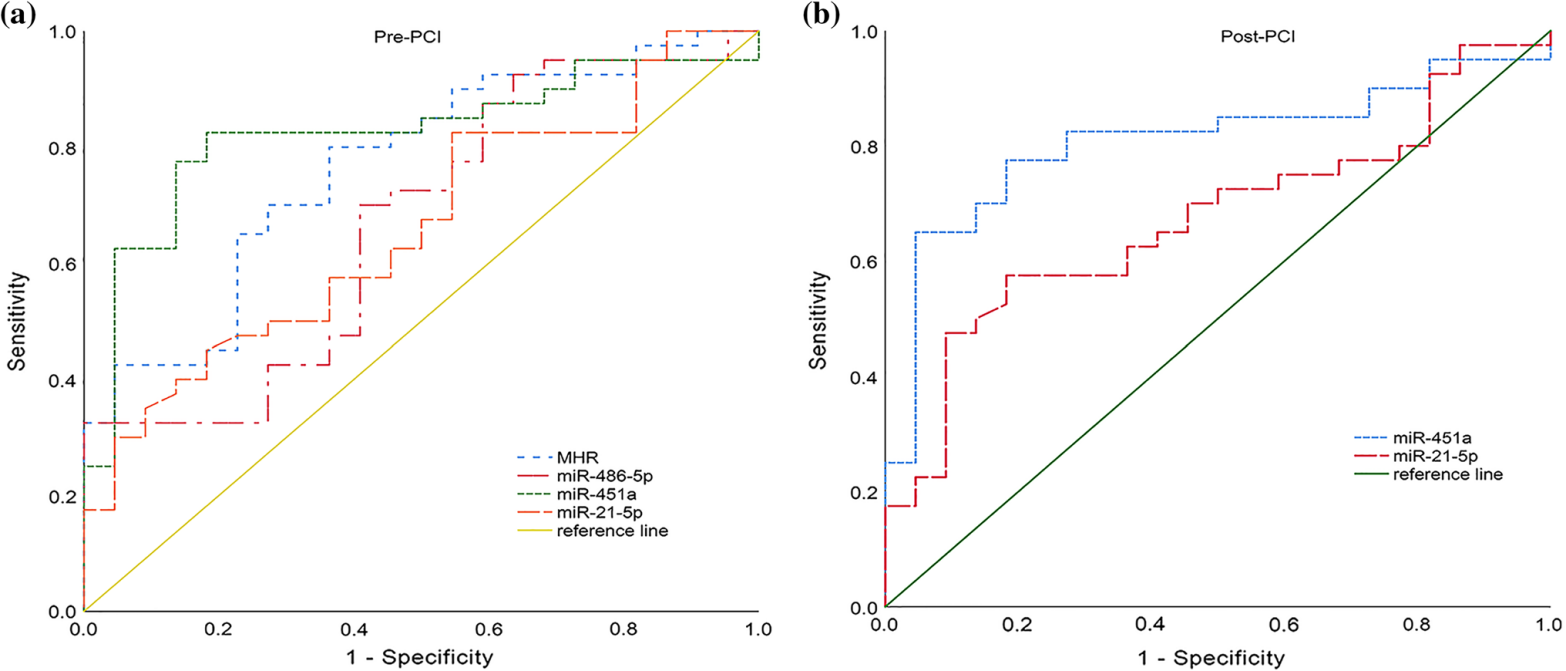
Receiver operating characteristic (ROC) curves analysis of miRNAs and MHR for the diagnosis in patients with AMI, including pre-PCI (**a**) and post-PCI (**b**)

**Fig. 4:**
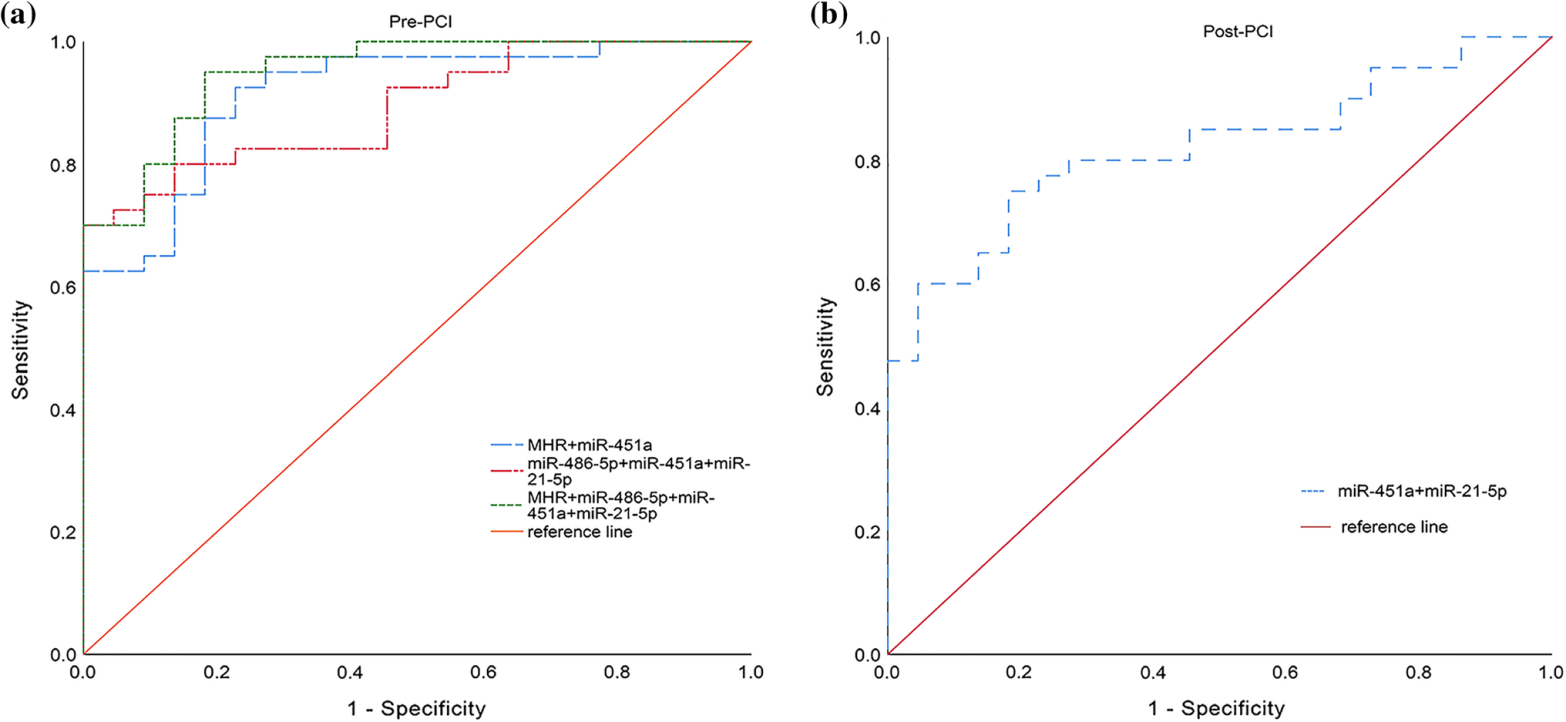
Receiver operating characteristic (ROC) curves analysis of the combination of miRNAs and MHR for predicting AMI, including pre-PCI (**a**) and post-PCI (**b**)

ROC curves were used to investigate the diagnostic value of these circulating miRNAs and MHR. As shown in Fig. 3a and Table 4, the area under the ROC curve before PCI in AMI was 0.766 (95% CI 0.641–0.864) for MHR, 0.669 (95% CI 0.538–0.783) for miR-486-5p, 0.823 (95% CI 0.706–0.908) for miR-451a and 0.660 (95% CI 0.529–0.776) for miR-21-5p. Next, we conducted the combined detection of these indicators (Fig. 4a and Table 4). Combined detection of MHR and miR-451a, the area under the curve (AUC) was 0.915 (95% CI 0.844–0.985) before PCI. When we combined detection of these miRNAs, AUC was 0.893 (95% CI 0.817–0.969) before PCI. Most interestingly, combined detection of all indicators, the AUC was 0.950 (95% CI 0.901–0.999) with sensitivity of 81.8.5% and specificity of 95.00%, which was higher than of these miRNAs alone. We also further investigated the values of miR-451a and miR-21-5p after PCI, which was 0.808 (95% CI 0.697–0.919) for miR-451a and 0.674 (95% CI 0.539–0.808) for miR-21-5p (Fig. 3b and Table 5). We conducted the combination of miR-451a and miR-21-5p, the results showed that the AUC was 0.82 (95% CI 0.702–0.906) after PCI with sensitivity of 75.0% and specificity of 81.80% (Fig. 4b and Table 5).

**Table 4:** Diagnostic value of miRNAs and MHR before PCI by ROC curve analysis

Index	AUC	95% CI	Specificity (%)	Sensitivity (%)	Youden index	Cut-off
MHR	0.766	0.641–0.864	63.60	80	0.436	> 0.343
miR-486-5p	0.669	0.528–0.810	70.00	59.10	0.291	≤ 0.065
miR-451a	0.823	0.706–0.908	81.80	82.50	0.643	> 0.062
miR-21-5p	0.660	0.529–0.776	45.50	82.50	0.280	≤ 0.026
MHR + miR-451a	0.915	0.844–0.985	77.30	92.50	0.698	–
miR-486-5p + miR-451a + miR-21-5p	0.893	0.817–0.969	95.50	72.50	0.680	–
MHR + miR-486-5p + miR-451a + miR-21-5p	0.950	0.901–0.999	95.00	81.80	0.768	–

**Table 5:** Diagnostic value of miR-451a and miR-21-5p after PCI by ROC curve analysis

Index	AUC	95% CI	Specificity	Sensitivity	Youden index	Cut-off
miR-451a	0.808	0.697–0.919	0.955	0.65	0.605	> 0.09
miR-21-5p	0.674	0.539–0.808	0.909	0.475	0.384	< 0.0065
miR-451a + miR-21-5p	0.82	0.702–0.906	0.818	0.75	0.568	–

### The relationship between miRNAs, MHR and cardiovascular risk factors

**Fig. 5:**
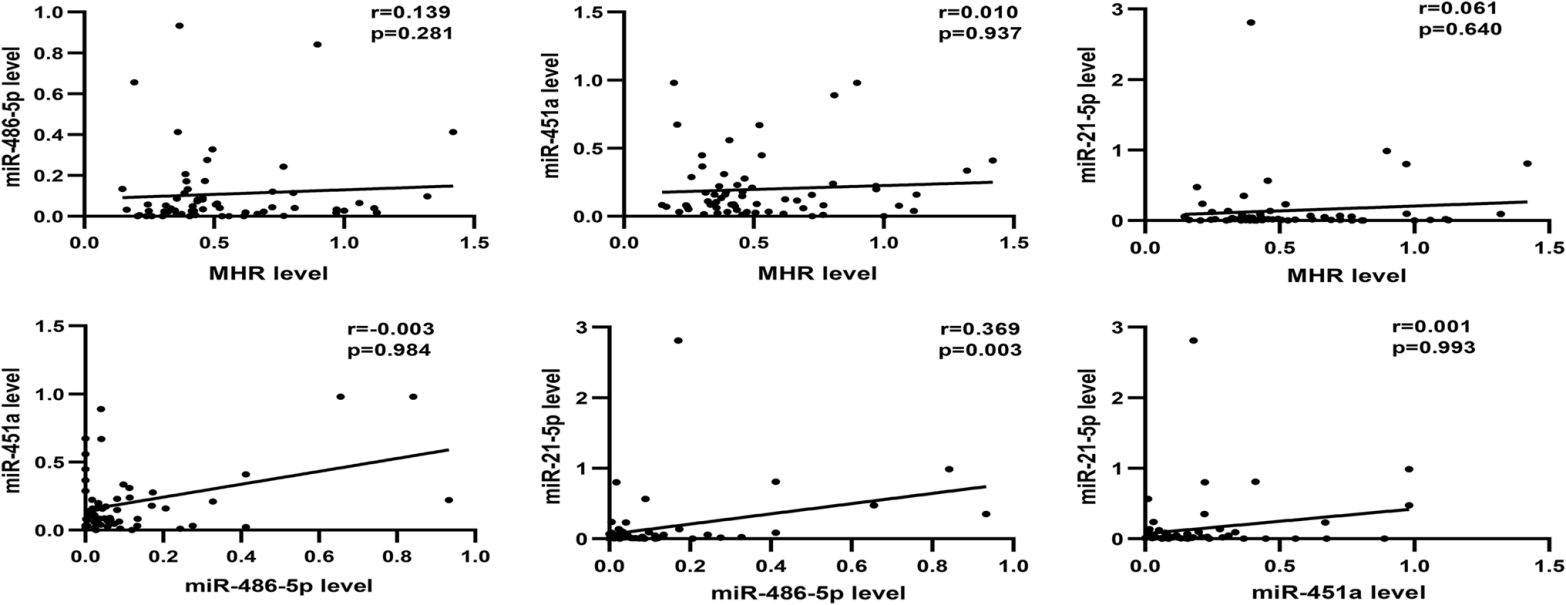
Spearman correlations between circulating miRNAs and MHR in AMI and UAP subjects

We analyzed the relationship between miRNAs and various indicators in AMI and UAP patients (Table 6). The expression of MHR was negatively correlated with diastolic blood pressure (*r* = − 0.277, *p* = 0.029) and HDL (*r* = − 0.541, *p* = 0.000). The level of miR-486-5p was positively correlated with TG (*r* = 0.355, *p* = 0.005) and negatively correlated with HDL (*r* = − 0.276, *p* = 0.030). The expression of the MHR was positively correlated with WBC (*r* = 0.309, *p* = 0.015). On the contrary, the level of miR-21-5p was negatively correlated with WBC (*r* = − 0.308, *p* = 0.015). Moreover, we analyzed the relationship between miRNAs and markers of myocardial injury, the results showed that the expression of miR-21-5p was negatively correlated with CK-MB (*r* = − 0.288, *p* = 0.023) and cTnI (*r* = − 0.411, *p* = 0.001) respectively. We further analyzed the relationship between these miRNAs and MHR in AMI and UA patients (Fig. 5). Interestingly, miR-21-5p was positively correlated with miR-486-5p (*r* = 0.369, *p* = 0.003). However, there was no correlation between MHR and miRNAs in AMI and UAP patients (Fig. 5a–c).

**Table 6:** Correlation analysis between miRNAs, MHR and classical cardiovascular risk factors

Variables	MHR		miR-486-5p		miR-451a		miR-21-5p	
	*r*	*p*	*r*	*p*	*r*	*p*	*r*	*p*
BMI	− 0.007	0.957	0.051	0.696	0.128	0.323	0.183	0.154
SBP	− 0.169	0.118	0.055	0.669	0.002	0.988	0.017	0.899
DBP	− 0.277	**0.029**	0.103	0.427	0.012	0.927	0.003	0.979
TG	0.115	0.372	0.355	**0.005**	0.017	0.898	0.168	0.191
TC	− 0.161	0.212	− 0.118	0.36	0.117	0.364	− 0.247	0.053
HDL	− 0.541	**0**	− 0.276	**0.03**	0.008	0.95	− 0.212	0.097
LDL	− 0.08	0.535	− 0.237	0.064	0.07	0.589	− 0.202	0.116
GLU	0.115	0.372	0.156	0.227	0.232	0.07	0.038	0.772
Cr	− 0.036	0.784	0.125	0.332	0.058	0.653	0.199	0.121
WBC	0.309	**0.015**	− 0.156	0.225	0.173	0.18	− 0.308	**0.015**
CK-MB	0.025	0.846	− 0.19	0.14	0.034	0.791	− 0.288	**0.023**
cTnI	0.192	0.136	− 0.124	0.335	0.003	0.984	− 0.411	**0.001**
N	0.232	0.07	− 0.147	0.256	0.138	0.285	− 0.233	0.069
L	0.273	**0.032**	0.195	0.129	0.066	0.609	0.054	0.676
M	0.819	**0**	− 0.009	0.945	0.027	0.834	− 0.017	0.894
PLT	− 0.166	0.197	− 0.074	0.57	− 0.133	0.302	− 0.105	0.416

*r* = Spearman rank correlation coefficients, *p* = *p*-value, values with statistical significance were indicated in bold

### Univariate and multivariate analysis

The results of the univariate analysis were shown in Table 7, these miRNAs and MHR were then incorporated into a progressive multivariate logistic regression model that included LDL, TC, TG, GLU and WBC. The results showed that the MHR and miR-486-5p were not independent risk factors before PCI in AMI. On the contrary, miR-451a (OR 9.041, 95% CI 1.345–60.762) and miR-21-5p (OR 0.134, 95% CI 0.027–0.667) were independent predictors before PCI. MiR-451a nor miR-21-5p were not independent risk factors after PCI in AMI.

**Table 7:** Univariate logistic regression and multivariate logistic regression analysis for the risk of AMI

Groups	Factor	Univariate logistic regression			Multivariate logistic regression		
		OR	95% CI	*p*-value	OR	95% CI	*p*-value
Pre-PCI	MHR	10.695	1.61–71.25	0.014	2.873	0.032–256.752	0.645
	miR-486-5p (log)	1	1–1.001	0.314	1	0.999–1.001	0.95
	miR-451a (log)	4.123	1.593–10.671	0.003	9.041	1.345–60.762	0.024
	miR-21-5p (log)	0.37	0.201–0.680	0.001	0.134	0.027–0.667	0.014
Post-PCI	miR-451a (log)	3.181	1.302–7.773	0.011	1.006	0.298–3.396	0.993
	miR-21-5p (log)	0.436	0.252–0.754	0.003	0.515	0.242–1.098	0.086

The model included LDL, TC, TG, GLU, WBC. OR were given for variation of every tenfold of miRNA

*CI* confidence interval, *OR* odds ratio

### Comparison in MACE and non-MACE groups

**Fig. 6:**
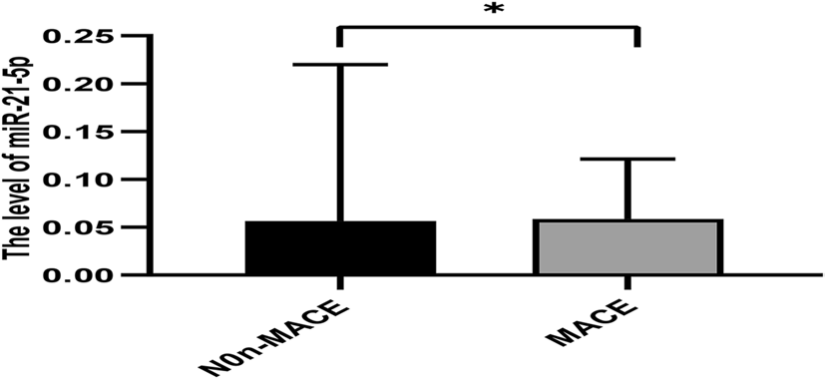
miRNA-21-5p level in the MACE and non-MACE groups before PCI (*p < 0.05)

**Fig. 7:**
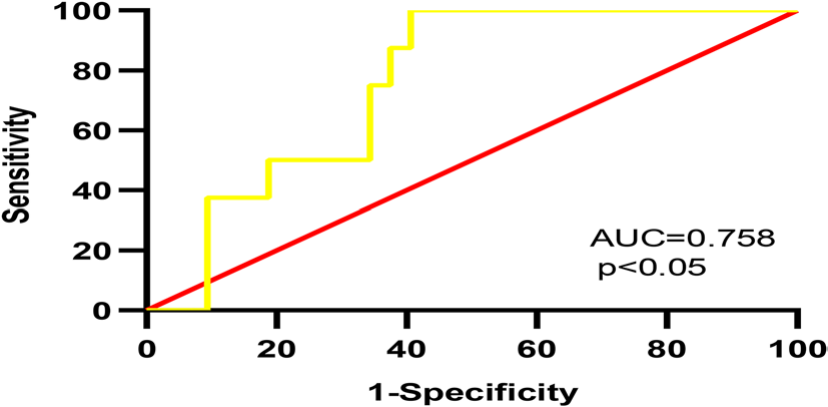
ROC curve analysis the predictive value of miR-21-5p for the occurrence of MACE before PCI

As shown in Table 8 and Fig. 6, we followed up the AMI group for 3 months, and then analyzed the significance of MHR and these miRNAs between the MACE group and the non-MACE group. The results showed that the level of miR-21-5p was decreased in the MACE group than in the non-MACE group (*p* < 0.05). We further analyzed the value of miR-21-5p in predicting the occurrence of MACE through the ROC curve, the result showed that the AUC was 0.758 (95% CI 0.608–0.908) for the miR-21-5p before PCI in AMI (Fig. 7). The remaining miRNAs and MHR had no statistical significance before and after PCI in the MACE group and the non-MACE group.

**Table 8:** Comparison in MCAE and non-MACE groups before PCI and after PCI

Groups	Variables	non-MACE	MACE	*p*-values
Pre-PCI	MHR	0.53 ± 0.28	0.66 ± 0.32	0.199
	miR-486-5p	0.10 ± 0.18	0.05 ± 0.06	0.521
	miR-451a	0.25 ± 0.25	0.24 ± 0.32	0.6
	miR-21-5p	0.06 ± 0.16	0.05 ± 0.06	0.026
Post-PCI	miR-451a	0.21 ± 0.21	0.24 ± 0.29	0.973
	miR-21-5p	0.14 ± 0.59	0.22 ± 0.29	0.112

### Bioinformatic analysis of miRNAs

**Fig. 8:**
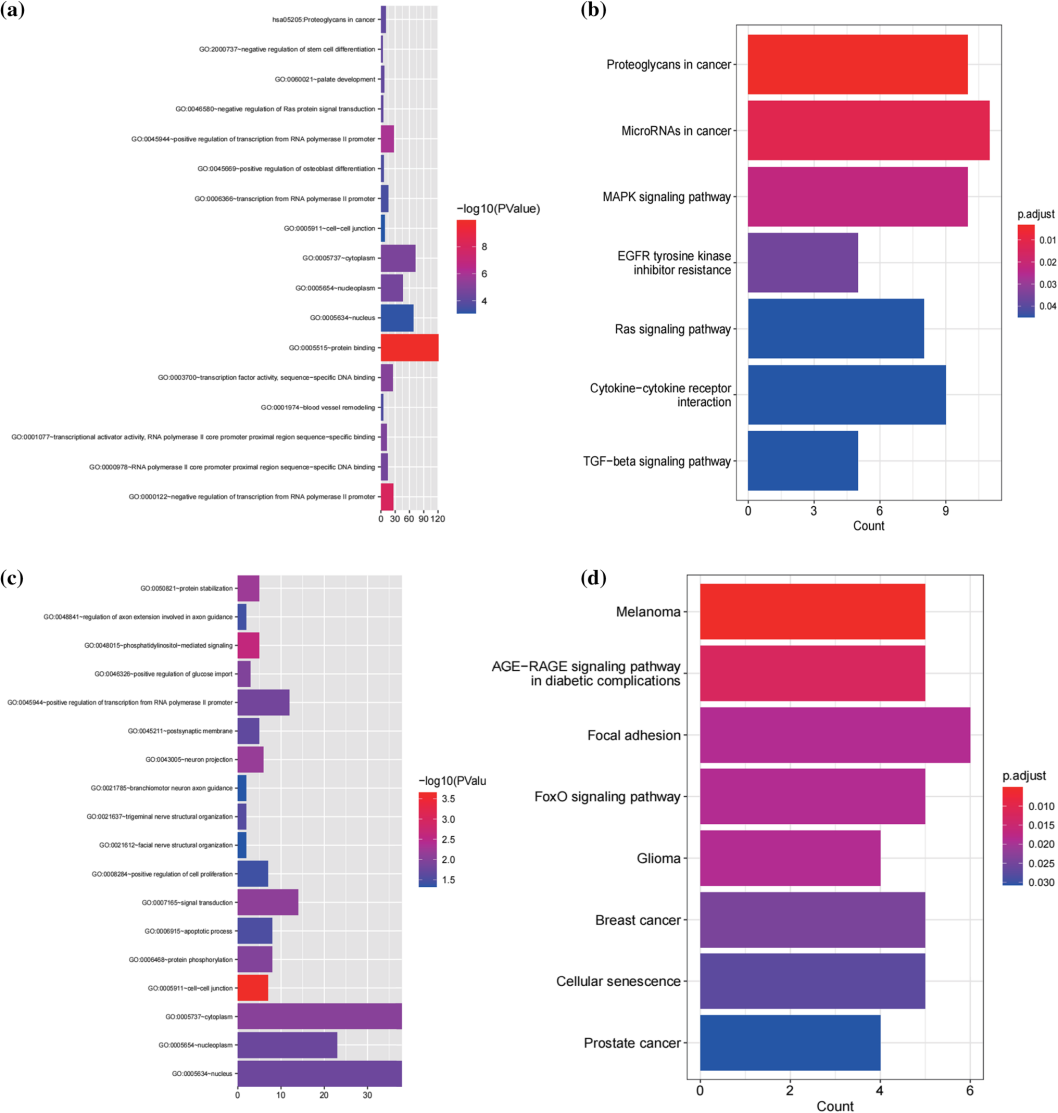
Bioinformation analysis of miRNAs

To further identify the function of candidate miRNAs in AMI, Go and KEGG enrichment analysis were performed for the selected target genes. The target genes of miR-451a were not significantly enriched in any term. The target genes of miR-21-5p were visibly enriched in terms such as protein binding, cytoplasm and proteoglycans in cancer (Fig. 8a, c). The target genes of Mir-486-5p were enriched in cell–cell junction, cytoplasm and FoxO signaling pathway (Fig. 8b, d). FoxO signaling pathway is closely related to oxidative stress, apoptosis and other pathophysiological processes which were closely related to AMI and ischemia injury. Therefore, we hypothesized that miR-486 is involved in AMI by regulating FoxO signaling pathway.

### miR-486 was downregulated in the AMI rats and hypoxia H9c2 cells

**Fig. 9:**
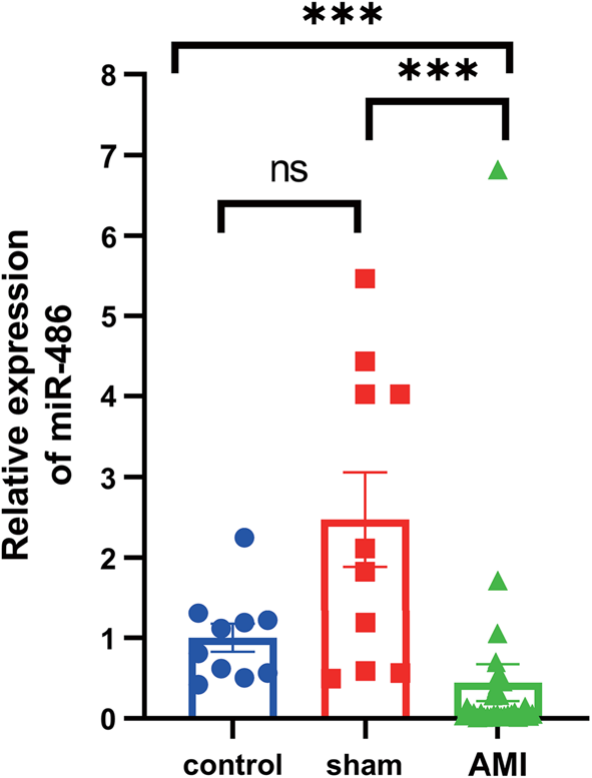
miR-486 was downregulated in the rat myocardium after AMI (*p < 0.05, **p < 0.01)

**Fig. 10:**
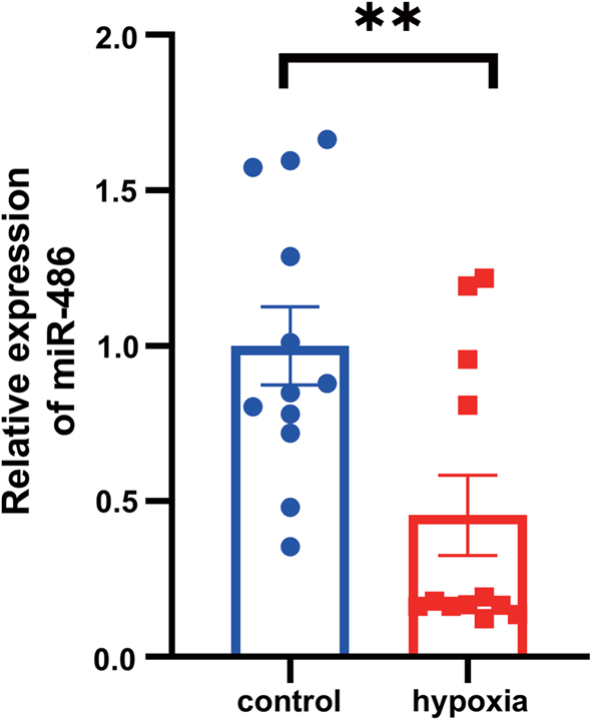
miR-486 was downregulated in H9c2 cells treated with hypoxia (*p < 0.05, **p < 0.01)

As mentioned above, among the candidate genes studied by our group, there are relatively more experiments on the mechanism of miR-21-5p and miR-451a. In addition, the interesting performance of miR-486-5p, especially its significantly different expression before and after PCI in the same patients, attracted our attention in the preconditioning experiment. In summary, we conducted a further study on miR-486. To verify the previous results, we simulated myocardial infarction in rats and H9c2 cells and detected the relative expression of miR-486. Compared to the control group, the expression of miR-486 declined dramatically in the AMI rats (Fig. 9). Similarly, H9c2 cells showed lower expression of miR-486 when they treated with hypoxia (Fig. 10).

## Discussion

AMI is a key component of cardiovascular disease, accurate and timely identification of the AMI is of great significance for reducing complications and improving long-term prognosis, especially for various isolated atypical symptoms of AMI [19]. In recent years, potential and non-invasive cardiovascular biomarkers have been received substantial research attention [20]. A large sequencing identified several miRNAs such as miR-1, miR-30d, miR-208, have high expression in healthy cardiac tissue [21]. Studies have shown that miR-208b and miR-499 were potential candidate biomarkers for AMI [22, 23]. It’s suggested that miRNAs play a pivotal role in cardiac development as well as disease pathophysiology.

As a new inflammatory parameter, MHR is a predictor of atherosclerotic development, progression, and predictor of clinical prognosis in CVD associated with inflammatory states [16]. MHR was decreased after PCI compared with before PCI. As De Rosa et al. said [24] that the increased transcoronary concentration gradients of miR-133a may be due to either a higher cardiac release or a reduced degradation of this miRNA during the transcoronary passage. Therefore, we speculated that MHR may have important significance in guiding myocardial reperfusion therapy by different miRNAs concentration patterns [25]. Balta et al. [26] concluded that MHR level may be used as an inflammatory marker for the prediction of no-reflow phenomenon among AMI patients undergoing PCI. Kanbay et al. [27] found that MHR could independently predict major cardiovascular events during follow-up. We observed the expression of MHR had no significant difference during follow-up, which might be associated with the small sample and the different response of patients with the disease. In addition, a follow-up study found that miR-21-5p could be used as a potential biomarker to guide the prognosis of AMI. There was a study have validated the function of miRNAs in MACE through patients with STEMI and found that miR-21-5p was lower in the MACE group than the non-MACE group [28]. Our result was consistent with Yang et al. More importantly, miR-21-5p was negatively correlated with cTnI. The results also demonstrated the clinical value of the indicators. Studies have shown that miRNAs are easier to detect than cTnI in patients with AMI during the earlier stage, indicating that miRNAs may respond to cell ischemia and hypoxia. However, cTnI was released into the blood during myocardial hypoxia and ischemia [29]. Thus, experts believe that the expression of miRNAs may be more significant in the early stage of AMI.

MiR-486-5p was found in solid malignancies, such as hepatocellular cancer, lung cancer, breast cancer, pancreatic cancer and so on [30]. Zhang et al. have demonstrated that over-expression of miR-486 alleviated hypoxia-induced myocardial injury in H9c2 cells, while suppression of miR-486 further aggravated hypoxia-induced injury. Zhang et al. [31] showed that the level of miR-486 was significantly higher in AMI than healthy controls, especially NSTEMI. Similarly, Wei et al. [32] agreed with the view and showed that miR-486-3p could distinguish STEMI and stable ischemic heart disease. In our study, our results suggested that miR-486-5p was a protective factor [9]. MiR-451 was associated with coronary plaque rupture and increased in the peripheral blood of patients with UA and AMI patients [33]. This was consistent with the results of our study. Further analysis showed that miR-451a had no statistical difference before and after PCI, which might be a special dynamic characteristic. Namely miR-451 was significantly decreased at 0.5 h after PCI induced plaque rupture and rapidly recovered in 1 h [34]. Our results also showed that miR-451a would be beneficial supplement of the MHR. Moreover, it has been reported that alterations of miR-21 may depend on AMI moment and specific location within the heart [35]. MiR-21was downregulated in infarcted areas of the heart, but upregulated in borderline areas [36]. Our results showed that miR-21-5p was decreased in the early stage of AMI. This indicating that miR-21-5p was released into the circulation in infarcted areas of the heart, while miR-21-5p might be detected from the borderline areas in the late stage of AMI. The differential modulation of miR-21-5p in the AMI may reflect pathophysiological mechanism in the progression and complications of AMI. Previous studies have demonstrated that miR-21 participates in the attenuation of inflammation or the angiogenic repair process of ischemic injury via NF-kappa B or PTEN/AKT/ERK1-VEGF/TGFβ2 pathway [37], and plays a cardiac protective role by mediating protein Period 2 (PER2) [38].

Many studies have demonstrated the potential of miRNAs as markers of CVD. For example, previously researchers have reported that miR-208a in plasma may be a novel biomarker for early detection of myocardial injury in humans [39]. We also confirmed that the levels of circulating miR-451a and miR-21-5p were associated with before PCI in AMI and could be used as independent predictors of AMI before PCI. MiR-451a showed the most accurate result among all indicators before PCI. Combined detection of the MHR and miRNAs, the AUC was 0.950 and 0.820, respectively, before PCI and after PCI in AMI patients, indicating that the combination of the miRNAs and MHR was more accurate than individual trait. Most importantly, miR-21-5p may be a potential marker for predicting the occurrence of MACE. Although they may not be better than traditional markers, these miRNAs could provide incremental value for the diagnosis of AMI and reflect pathophysiological mechanism in the progression and complications of AMI.

There are still some limitations to our research. Firstly, we are unable to record the first time of onset of AMI patients. With the development and changes of the occurrence of AMI, circulating miRNAs will change. Secondly, we recruited relatively small samples to analyze miRNAs. To make our results more accurate, additional investigations with larger cohorts of healthy people and patients are needed in the next experiment. The study should be primarily viewed as a proof-of-concept study discussing potential mechanism of circulating miRNAs in the AMI. In the future, we will carry out dynamic and continuous monitoring of the expressions of miRNAs and cTnI. Finally, more further studies on the mechanism of action of miRNA are needed to determine its role in AMI.

## Conclusions

In summary, we found that the dynamic expressions of the miRNAs and MHR of the patients with AMI. ROC analysis suggested that circulating miR-486-5p, miR-451a, miR-21-5p and MHR may play critical roles in the early phase of AMI, and may be used as potential predictors for diagnosis. MiR-451a was a more reliable biomarker in diagnosing AMI. The results suggested that miRNAs played important roles in the pathophysiology of AMI. Most interesting, miR-21-5p might predict long-term MACE events after AMI. In addition, miR-486 may participate in AMI by regulating FoxO signaling pathway.
